# Genome Sequence of *Bacillus anthracis* STI, a Sterne-Like Georgian/Soviet Vaccine Strain

**DOI:** 10.1128/genomeA.00853-14

**Published:** 2014-09-18

**Authors:** Richard T. Okinaka, Jean Challacombe, Kevin Drees, Dawn N. Birdsell, Nicolette Janke, Amber Naumann, Meagan Seymour, Heidie Hornstra, James Schupp, Jason Sahl, Jeffrey T. Foster, Talima Pearson, Peter Turnbull, Paul Keim

**Affiliations:** a Center for Microbial Genetics and Genomics, Northern Arizona University, Flagstaff, Arizona, USA; b Bioscience Division, Los Alamos National Laboratory, Los Alamos New Mexico, USA; c Pathogen Genomics Division, Translational Genomics Research Institute, Flagstaff, Arizona, USA; d Centre for Applied Microbiology and Research, Porton Down, Salisbury, United Kingdom

## Abstract

The *Bacillus anthracis* strain STI is a Soviet vaccine strain that lacks the pXO2 plasmid. Previous data indicate that this isolate forms a new branch within the *B. anthracis* sub-group originally identified as A. Br.008/009.

## GENOME ANNOUNCEMENT

Two distinct Soviet anthrax vaccine strains have been previously described. The first is a rare isolate that was sequenced by the J. Craig Venter Institute (JCVI) and is known as *Bacillus anthracis* strain Tsiankovskii-I (accession no. PRJNA54481). The GenBank entry for this genome indicates that this particular strain was used as an animal vaccine and is unusual because it contains both of the virulence plasmids of *B. anthracis*, pXO1 and pXO2. The second Soviet anthrax vaccine strain is the subject of this Genome Announcement. This strain is the more publicized Georgian/Soviet vaccine strain known as STI (1–4). The original vaccine was purportedly developed by Soviet Scientists in the 1940s at their Kirov Institute and was successfully used to vaccinate animals (2). A similar human anthrax vaccine of STI was produced in the Vaccine/Serum Institute, Tbilisi, Georgia (3, 4). This is a Sterne-like vaccine strain that lacks the pXO2 plasmid that accounts for the production of a poly-glutamate capsule layer, one of the virulence factors that define *B. anthracis*. Previous studies indicated that both of these isolates are on a specific sub-branch of *B. anthracis* that has been defined as the trans-Eurasian cluster (also A. Br.008/009) so named because the vast majority of >200 isolates from this sub-group in the *B. anthracis* collection at Northern Arizona University, were originally geo-located throughout Europe and China (5–7). The genome of the STI strain is therefore, distinct from the original Sterne vaccine strain and it is part of a new paraphyletic branch within the trans-Eurasian cluster. The precise strain that was sequenced has a designation BaA0485 in the Keim Laboratory archive and is also designated as STI-89 and K2789. This strain was provided by P. Turnbull.

Whole-genome shotgun sequencing (WGS) of the STI isolate (A0485; aka STI-89, K2789) was performed using the Illumina GAIIx sequencing platform. Following fragmentation to 500 bp insert sizes, end reparation, and sample tagging, the sequencer produced 4.45 million (genome) and 495,514 (pXO1 plasmid), 100-bp paired-end reads or coverage data at 87× and 256× for the genome and the pXO1 plasmid, respectively. Comparative assemblies were performed with both DNASTAR and CLC Bio Genomics Workbench (http://www.clcbio.com/) using the Ames ancestor reference genome (6) as a template. The conserved nature of the *B. anthracis* genome and the standard use of the Ames ancestor reference genome greatly facilitate the identification of unique regions in new assemblies. A *de novo* assembly was also performed using the paired-end sequence data with CLC Bio Genomics WorkBench and the resulting analysis yielded 126 contigs. Both the template and *de novo* assembled contigs were submitted to the RAST annotation server for subsystem classification and functional annotation (http://metagenomics.anl.gov/) (8).

### Nucleotide sequence accession numbers.

The NCBI BioProject ID is PRJNA243052 and the GenBank accession numbers for this STI genome are BaA0485_chr (CP007660) and BaA0485_pXO1 (CP007661).

## References

[1] Donegan S Bellamy R Gamble CL 2009 Vaccines for preventing anthrax. *In* Cochrane Database of Systematic Reviews 2009. Wiley, New York. 10.1002/14651858.CD006403.pub2 PMC6532564 19370633

[2] Leitenberg M Zilinskas RA Kuhn JH 2012 The Soviet Biological Weapons Program, vol 1 Harvard University Press, Cambridge, MA

[3] Turnbull P 2010 Anthrax vaccines, p 57–71 *In* Artenstein AW (ed), Vaccines: A Biography. Springer Verlag, London

[4] Turnbull PC 1991 Anthrax vaccines: past, present and future. Vaccine 9:533–539 1771966 10.1016/0264-410x(91)90237-z

[5] Pearson T Busch JD Ravel J Read TD Rhoton SD U’Ren JM Simonson TS Kachur SM Leadem RR Cardon ML Van Ert MN Huynh LY Fraser CM Keim P 2004 Phylogenetic discovery bias in *Bacillus anthracis* using single-nucleotide polymorphisms from whole-genome sequencing. Proc. Natl. Acad. Sci. U. S. A. 101:13536–13541. 10.1073/pnas.0403844101 15347815 PMC518758

[6] Simonson TS Okinaka RT Wang B Easterday WR Huynh L U’Ren JM Dukerich M Zanecki SR Kenefic LJ Beaudry J Schupp JM Pearson T Wagner DM Hoffmaster A Ravel J Keim P 2009 *Bacillus anthracis* in China and its relationship to worldwide lineages. BMC Microbiol. 9:71. 10.1186/1471-2180-9-71 19368722 PMC2674057

[7] Van Ert MN Easterday WR Huynh LY Okinaka RT Hugh-Jones ME Ravel J Zanecki SR Pearson T Simonson TS U’Ren JM Kachur SM Leadem-Dougherty RR Rhoton SD Zinser G Farlow J Coker PR Smith KL Wang B Kenefic LJ Fraser-Liggett CM Wagner DM Keim P 2007 Global genetic population structure of *Bacillus anthracis*. PLoS One 2:e461. 10.1371/journal.pone.0000461 17520020 PMC1866244

[8] Aziz RK Bartels D Best AA DeJongh M Disz T Edwards RA Formsma K Gerdes S Glass EM Kubal M Meyer F Olsen GJ Olson R Osterman AL Overbeek RA McNeil LK Paarmann D Paczian T Parrello B Pusch GD Reich C Stevens R Vassieva O Vonstein V Wilke A Zagnitko O 2008 The RAST server: Rapid Annotations using Subsystems Technology. BMC Genomics 9:75. 10.1186/1471-2164-9-75 18261238 PMC2265698

